# Spatial multi-omics characterizes neuronal mimicry as a coherent hybrid state in glioblastoma

**DOI:** 10.1093/bib/bbaf631.034

**Published:** 2025-12-12

**Authors:** Claire Yuan, Jake Y Chen

**Affiliations:** AlphaMind Club, Birmingham, AL, United States; AlphaMind Club, Birmingham, AL, United States; Systems Pharmacology AI Research Center, School of Medicine, The University of Alabama at Birmingham, Birmingham, AL, United States

## Abstract

**Aim:**

Glioblastoma (GBM) exhibits pronounced cellular plasticity that contributes to therapeutic resistance. A facet of this adaptability is neuronal mimicry, whereby tumor cells acquire neuron-like features to engage with neural circuits. The coherence and biological significance of this process remain uncertain.

**Methods:**

Spatial transcriptomic and proteomic datasets from Greenwald et al. [1] were analyzed alongside healthy cortical tissue from Ravi et al. [2] as a non-malignant reference. A permutation-based framework identified tumor–neuronal (TN) cells across Healthy, Core, Infiltrative, and Bulk regions, with stability verified by resampling and Jaccard overlap (CV < 10%).

**Results:**

TN cells were enriched in Bulk (OR ≈ 70, p < 10^−10^) and Infiltrative (OR ≈ 7, p < 10^−10^) regions relative to Healthy. Integrative analyses revealed a synaptic-like transcriptional program marked by SNAP25, SYT1, and STMN2 (logFC ≈ 1–1.5), enriched for vesicle cycling and cytoskeletal remodeling pathways. Neuronal subtype markers (SLC17A7, GABRA1) were depleted, while malignant markers (CLU, GFAP, VIM) persisted. Single-cell proteomics confirmed co-localization of neuronal and tumor proteins within clustered, double-positive cells.

**Conclusion:**

This study delineates a reproducible hybrid state with partial neuronal characteristics, suggesting that neuronal mimicry represents an adaptive, rather than lineage-converted, trajectory of GBM that may inform strategies to disrupt tumor–neuron crosstalk.

**References:**

1. Greenwald AC, et al. ‘Integrative spatial analysis reveals a multi-layered organization of glioblastoma.’ *Cell* 2024;187(10):2485–2501.e26.

2. Ravi V, Will P, Kueckelhaus J, et al. ‘Spatially resolved multi-omics deciphers bidirectional tumor-host interdependence in glioblastoma.’ *Dryad* 2025;10.5061/dryad.h70rxwdmj.

